# Supplementary material for: Adaptations of data mining methodologies: a systematic literature review
Source: PeerJ Comput Sci. 2020 May 25;6:e267. doi: 10.7717/peerj-cs.267 (PMC7924527; doi:10.7717/peerj-cs.267)
Supplement: Supplemental Information 1 — Unfortunately, we were not able to upload any graph (original png files). Based on Overleaf placed PeerJ template we constructed graphs files based on the template examples. Unfortunately, we were not able to understand why it did not fit, redoing to new formats will change all texts flow and generated pdf file. We submit graphs in archived file as part of supplementary material. We will do our best to redo the graphs further based on instructions from You. [file peerj-cs-06-267-s001.pdf]

Systematic Literature Review Protocol: Adaptations of Data Mining Methodologies

Veronika Plotnikova, Marlon Dumas, Fredrik P.Milani

University of Tartu, Institute of Computer Science, J. Liivi 2, 50409 Tartu, Estonia
name.surname@ut.ee

Protocol version	Version released	Content or update summary	Update date	Update done by
Version 0.1.	5/08/2018	First draft version of protocol to guide the research process	-	V. Plotnikova
Version 0.2	12/09/2018	Update to key research design elements (based on supervisors comments)	19/09/2018	V. Plotnikova
Version 0.3	17/11/2018	Release of new version based on work with text corpus, update based on results of research	20/05/2019	V. Plotnikova
Version 0.4	5/11/2019	Release of new version based on Revised Manuscript as well as Reviewers' and Editor's suggestions	-	V. Plotnikova

1 Introduction

The 'Big Data' phenomenon, technological advances in data processing and development of algorithmic techniques have fostered widespread adoption of data analytics across different industries. According to the most recent market studies [1]-[2] adoption rate of 'Big Data' analytics tripled for all companies reaching 53% in 2017, up from 17% in 2015.

Telecommunications and financial services are the leading industry adopters with 87% and 76% of the respective sector companies already reporting the data analytics usage – well above average figures. They have developed specific datasets, varieties of data and execute broad set of data mining tasks to solve industry-specific business problems. Therefore, both industries are naturally the most suitable sectors for in-depth exploration of data analytics phenomena and its impact on organizations and business practices. Also, both telecoms and financial services explicitly demonstrate the trend of heavy investments into data analytics technologies and competences seeking to realize benefits from data-driven decision-making and maximize 'Big Data' business value.

However, 'Big Data' and Advanced Analytics projects failure rates are extremely high – according to recent 2017 estimates [3] at least 60% of the project

fail to realize business value. Many of the projects do not perform due to lack of knowledge on how to approach and tackle complex data analytics projects thus missing comprehensive, domain-specific methodological support.

The authors of the protocol have initiated PhD research project that aims to develop such practical support. The envisaged outcome is domain-specific reference framework that would assist practitioners in framing and conducting complex data mining and data analytics projects. As telecoms and financial services are identified as one of the most suitable sectors for in-depth exploration of data analytics business practices, the new framework will be designed for one of them - banking domain [1].

This protocol illustrates how Systematic Literature Review will be conducted as part of the given PhD project. Section 2 introduces research context in details and outlines purpose of the review. Section 3 presents research objectives and questions. Section 4 discusses the method that will be applied for Systematic Literature Review detailing the scope, search strategy, relevance and quality criteria and screening process. Section 5 outlines validation procedures while Section 6 concludes.

2 Background and Purpose

Given PhD research project consists of 3 key phases discussed in details below:

- Phase 1- exploration and consolidation
- Phase 2 - retrospective evaluation
- Phase 3 - application and refinement

In Phase 1 (Explorations and Consolidation) Systematic Literature Review (abbreviated SLR) is the primary research method used. Firstly, comprehensive data collection on existing data mining and data analytics methodologies, frameworks [2] and its consolidation will be performed. Secondly, analysis and synthesis of the existing knowledge base will serve as central input towards construction of the draft domain-specific data mining and data analytics reference framework.

Phase 2 (Retrospective Evaluation) of the project will be conducted as multiple case study (cross-case study). It will validate Phase 1 proposed framework by back-testing on the real life, diverse use cases portfolio; the outcome of this phase is refined version of framework.

After validation by means of multiple case-study, the relevance and utility of the framework is to be tested in the final Phase 3 of the project. It will be

[1] Hereinafter, the term *banking domain* refers to: (1) traditional businesses providing universal banking and insurance products and services (eg. lending, transactions, capital markets, asset management, etc.) to all types of clientele (private, corporate, financial institutions and firms), and (2) niche players, disruptors (FinTech, monoline banks etc.) specialized in specific banking, insurance products and services.

[2] Hereinafter, the research scope is limited to data mining and data analytics methodologies and frameworks. Such delimitation is discussed and motivated in details in subsection 4.1 as well as 5.1.

conducted by means of action research and will address validity, relevancy and most importantly utility of the proposed framework by applying it in real life data mining project.

3 SLR Objectives and Process

3.1 Research Objectives

This section addresses the objectives of Systematic Literature Review, associated research questions as well as process.

The primary objective of SLR is to analyze and systematize existing scientific knowledge concerning application of data mining and data analytics methodologies. In-depth examination of such application practices and consolidated knowledge will support the next research step - elicitation of customized, adapted, domain-specific draft reference framework for complex data mining and data analytics projects in financial services industry.

The SLR research objective is addressed in multi-step process by postulating and providing answer to the following research questions:

Research Question 1: How data mining methodologies are applied? - this question aims to identify data mining methodologies application and usage patterns and trends

Research Question 2: How have existing data mining methodologies been adapted? - this questions aims to identify and classify data mining methodologies adaptation patterns and scenarios

Research Question 3: For what purposes have existing data mining methodologies been adapted? - this question aims to identify, explain, classify and produce insights on what are the reasons and what benefits are achieved by adaptations of existing data mining methodologies. Specifically, what gaps do these adaptations seek to fill and what have been the benefits of these adaptations.

Based on this research scope, data extraction will be executed for RQ 1 while RQ 2 and RQ 3 will be addressed by analysis and synthesis of selected publications.

3.2 SLR Plan

Systematic Literature Review will be conducted based on best practices. The process is exhibited in Figure 1 below.

Systematic Literature Process consists of the 3 phases: (1) review planning, (2) review execution, and (3) review documentation phases. This protocol is an integral part of this project SLR and addresses Planning Phase. The purpose of this protocol is two-fold. Firstly, to clearly define and motivate Systematic Literature Review boundaries defining scope, selection of data sources, relevancy and quality-based exclusion/inclusion criteria, etc. thereby constructing well-defined, consistent decision rules for Systematic Literature Review execution.

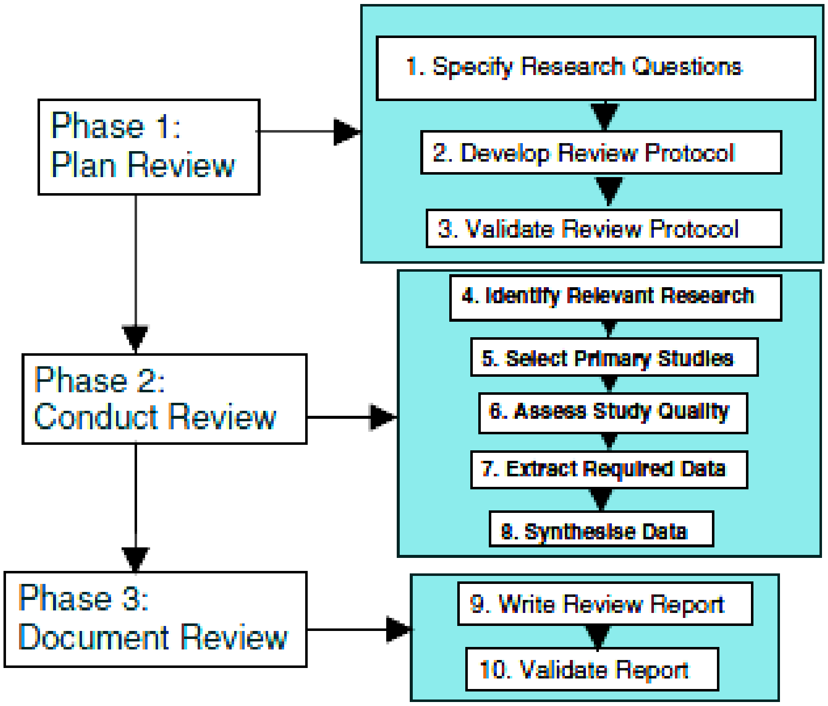

Fig. 1. Systematic Literature Review Process [5].

Secondly, to explicitly design and document envisaged review method and process ensuring traceability, transparency and study replicability. Apart from this, based on recommendations and best practices (eg.[5]), authors performed validation procedures by intensively piloting the protocol. Therefore, even if the study is formally has just completed planning phase, most of the elements required to conduct review are already in place or have been thoroughly piloted and explored.

4 Method

This section of the protocol addresses key elements of the SLR such as scope, search strategy, and publications screening principles.

4.1 Scope

Subject matter of this PhD research is methodological support for complex data analytics and data mining projects. Therefore, the scope of the research are existing methodologies and frameworks.

Methodology represents higher level of abstraction as system, set of methods, principles and rules[3] while method is defined as particular procedure[4] thus being subordinate to methodology. Therefore, methodology is identified as central element in research scope while data mining methods are encompassed indirectly under methodology umbrella. Authors have executed extensive pilots to validate the scope, using both "methodology" and "method" in the search strings (please refer to the detailed piloting results in section *5 Validation*, subsection *5.1.*) Both the enormous amount of texts received when running the search (well above 60 thousands), and sampled texts content analysis confirmed overwhelming application of the method as granular procedure. Therefore, methodology is confirmed to be primary scope to achieve objectives of this research and SLR.

Further, this PhD research project is guided by design science paradigm of Information Systems research. Design science paradigm in contrast to behavioral paradigm[5] creates and applies new and innovative artifacts to achieve knowledge and understanding of problem domain [8]. Based on this premise, existing conceptual and theoretical data mining and data analytics frameworks are the other central element in the scope.

What is explicitly out of scope and not intended to be investigated are publications, scientific texts which as research objective explore and consider:

1. context of technology and infrastructure for data mining/data analytics tasks and projects
2. granular methods, techniques to utilize in data mining process itself or apply for data mining tasks, eg. constructing business queries or applying regression or neural networks modeling techniques to solve classification problems
3. granular technological aspects, tasks in data mining eg. data engineering, dataflows and workflows
4. traditional statistical methods not associated with data mining directly including statistical control methods.

Abovementioned focus is motivated by the design science approach of the research as well as the fact that both data mining and data analytics are rather complex and comprehensive topics widely investigated and discussed in academia and among practitioners. As confirmed by extensive piloting of protocol (please refer to Section *5 Validation*), methodologies and frameworks is vast research domain with fragmented, cross-disciplinary knowledge base. Therefore, clear delimitation is necessary to succeed in consolidation of existing academic evidence and further creation of useful, valid and relevant artifacts (reference framework). As a result, tackling associated or interdisciplinary topics and themes is largely not possible within one doctoral project and could be addressed in the separate future research.

[3] Oxford English Dictionary, Cambridge Dictionary.
[4] Ibid.
[5] The behavioral science paradigm seeks to develop and verify theories that explain or predict human or organizational behavior [8].

4.2 Search Strategy

Types of Literature and Selection of Electronic Databases The purpose of this Systematic Literature Review is to comprehensively encompass existing body of knowledge. It includes both "peer-reviewed"/academic and partially non-peer-reviewed/industry (so-called "grey") literature. The decision to cover "grey" literature[6] in this research has been motivated by the following considerations. As proposed in number of information systems, software engineering domain publications (for example in [12] - [13], [14]), SLR as stand-alone method may not provide insights into "state of practice". Further, authors of [12] reported growing importance of secondary studies coupled with pronounced, growing trend of publishing various types of "grey" literature. Therefore, adapted types of secondary studies (eg. Multivocal Literature Reviews or MLR) which cover "grey" literature have emerged too and are now applied with growing confidence. It was also identified (eg. in [12]) that "grey" literature can give substantial benefits in certain areas of software engineering. Also, it was discovered that when numerous practical evidence was ignored (in case of academic only SLRs), it had produced profound negative impact on research directions. [12]-[13] demonstrated that with MLR method (i.e. with inclusion of "grey" literature) there were significant information gains and a lot of expertise could have been missed otherwise. Further, MLRs benefits have been reported also in other domains (eg. in education science) and even back in 1991. It should be noted that MLRs benefits are most pronounced when topic of research is related to industrial, practical settings.

Having studied typical reviews methodologies adapted for the usage of "grey" literature - such as Grey Literature Mapping, Grey Literature Review, Multivocal Literature Mapping, Multivocal Literature Review ([13])-and taking into consideration the research objectives which focus on investigating data mining methodologies application practices, we have opted for inclusion of elements of Multivocal Literature Review (MLR) in our study. As defined in [13], a Multivocal Literature Review (MLR) is a form of a Systematic Literature Review (SLR) which includes the grey literature (e.g., blog posts, videos and white papers) in addition to the published (formal) literature (e.g., journal and conference papers). Based on MLR practice, we have chosen to include both type of literature further denoted as "peer-reviewed" and "grey", however, treat them strongly separately. By inclusion of "grey" literature, we have also followed [4] recommendations whereby its inclusion supports minimizing publication bias due to the fact that positive results and research outcomes are more likely to be published than negative ones. Following MLR practices, we also designed inclusion criteria for types of "grey" literature, and they are reported below.

[6] We rely on classical, widely used Luxembourg definition of "grey" literature. According to it: "[this] is literature produced on all levels of government, academics, business and industry in print and electronic formats, but which is not controlled by commercial publishers. New York definition adds clarification "i.e., where publishing is not the primary activity of the producing body" [11]

There are three databases selected to cover two types of literature: (1) indexed scientific databases (Scopus and Web of Science) as ”peer-reviewed” literature source, and (2) non-indexed database Google Scholar as source of ”grey” literature.

Selection of indexed scientific publications databases Scopus and Web of Science which contain academic research in the field was executed as follows. Firstly, we have evaluated multidiscplinary vs domain oriented databases. The broad target list of electronic databases consisted of 3 multidisciplinary-oriented (Scopus, Web of Science, Wiley Online Library) and 2 computer science domain oriented (ACM Digital Library, IEEE Xplorer Digital Library) electronic databases. We noted that domain-oriented databases ACM and IEEE while being comprehensive are focused on complete collection of respective institutions publications (Association for Computing Machinery, Institute of Electrical and Electronic Engineers respectively) and cover computer science and engineering areas. At the same time, significant amount of work in our targeted research field is performed in other domains. Therefore, multidisciplinary databases are preferred due to wider domain coverage. This choice was also supported by running pilot searches - it was confirmed that Scopus database search retrieves in significant proportions ACM and IEEE publications therefore, indirect coverage of ACM and IEEE texst is ensured. Secondly, we proceeded with evaluating multidisciplinary databases themselves. Scopus database coverage is unrivaled with abstracting more than 15 000 scientific journals which by publisher's assessment makes it world's largest database of peer-reviewed literature covering app. 80% of all international peer-reviewed journals. Web of Science is similar to Scopus, but covers longer temporal range. Thus, both databases complement each other. Wiley Online while hosting one of the world's most extensive multidisciplinary collections focuses on life, health and physical sciences, social science, and the humanities and for comparison covers only 1 500 journals, these facts imply less balanced domain view and lower coverage compared to Scopus. Pilot search confirmed that Wiley publications are also extensively indexed by Scopus and retrieved. Thus, out of 3 multidisciplinary databases initially evaluated, we have chosen for our SLR to use Scopus and Web of Science.

Selection of non-indexed, ”grey” literature source database was executed as follows. The selected database source is Google Scholar and it is not indexed, however, it is comprehensive source of both academic and ”grey” literature publications and referred as such extensively (eg.[14]-[15]). Also, there are many types of ”grey” literature, eg. [15] reported 22 such types while [14] adapted ”grey” literature classification to software engineering domain and categorized 7 types. We have been guided by [14] adaptation framework in our ”grey” literature inclusion criteria. However, we based them not only on type of ”grey” literature, but also considered classification of ”grey” literature producers. The latter is important given naturally limited control over expertise and origin of ”grey” literature. Therefore, we have used combined criteria considering type of literature jointly with producer. From the list of producers ([14] we have adopted and focused on: (1) Government departments and agencies (i.e., in municipal, provincial, or na-

tional levels); (2) Non-profit economic, trade organizations (”think-tanks”) and professional associations; (3) Academic and research institutions; (4) Businesses and corporations - consultancy companies and established private companies (not start-ups). Further, [14] presented three-tier categorization framework for types of ”grey literature”. In our study we restricted ourselves to the 1st tier ”grey” literature publications of the abovementioned producers, the selected items list include:

– government, academic, and private sector consultancies reports (including white papers, market reports, industry overviews and similar)
– theses (not lower than Master level) and PhD Dissertations
– research reports
– working papers
– conference proceedings, preprints.

With inclusion of the 1st tier ”grey” literature criteria we mitigate quality assessment challenge especially relevant and reported for it (see for example [13]-[14]). As observed, in contrast to scientific literature, ”grey” one includes studies from diverse several sources with differing, non-comparable quality. Thus, we have intentionally excluded from scope such literature producers as societies, parties, libraries, freelance individuals as well as 2nd and 3rd Tier literature types which is audio-video content, newsletters, bulletins, academic courseware, lecture notes and presentations, patents and similar. Further, with strict delimitation to the 1st Tier literature only, originated by the limited number of reliable producers, our intention is to capture only publications presenting and discussing the research topic and reporting on ”state of practice”. Literature which objective is not to provide such in-depth discussions and insights is left out.

Search terms and strings definition The search terms are derived from the research scope and respective research questions. They have been determined via extensive, iterative piloting and validation procedures of protocol search keywords and corresponding strings which is documented in details in Section 5 Validation.

To highlight key steps, initially, there were six separate search strings combinations:

1. (”data mining”) AND (”method”)
2. (”data mining”) AND (”methodology”)
3. (”data mining”) AND (”framework”)
4. (”data analytics”) AND (”method”)
5. (”data analytics”) AND (”methodology”)
6. (”data analytics”) AND (”framework”)

After piloting ans scoping activities, there were two final ancillary elements identified, i.e., ”methodology” and ”framework”.

Finally, there have been four search strings constructed:

1. two search strings as combinations of keyword ”data mining” with respective ancillary element:
   - (”data mining methodology”),
   - (”data mining framework”).
2. two search strings as combinations of keyword ”data analytics” with respective ancillary element:
   - (”data analytics methodology”),
   - (”data analytics framework”).

4.3 Quality and relevance screening

Based on the best Systematic Literature Review practices ([4]-[5]), authors have developed relevance screening criteria and procedure.

Exclusion and Inclusion criteria The initial quality screening and relevance assessment will be conducted after final data extraction executed in accordance with search strategy described and confirmed with validation procedures. It is expected to obtain significant number of publications, therefore, it is critical to define clear and comprehensive criteria to form encompassing and unbiased text corpus. Authors constructed two-tier relevance and quality criteria decision tree which has been also validated (please refer to section 5 Validation, especially subsections 5.2 and 5.4).

First-tier exclusion criteria are initial threshold quality controls aiming at eliminating the studies with little scientific contribution, limited or no topic discussion. Also, they address issues of availability, accessibility and English as primary language for publications. *First-tier exclusion criteria* are formulated as follows:

1. the publication item is not in English - understandability might not be achieved and contribution of the paper will be limited if accomplished at all.
2. the paper is not accessible in full length online through the university subscription of databases and via Google Scholar - not full availability limits analysis and any potential contribution.
3. publication item duplicates which can occur when:
   - either the same document retrieved in our case in two or all three databases - decision rule to retain one item in the final text corpus.
   - or different versions of the same publication are retrieved (i.e. the same study published in different sources) - based on best practices, decision rule is that the most recent paper is retained as well as the one with the highest score [9].
   - if a publication is published both as conference proceeding and as journal article (with the same name and same authors and extended), the latter is selected.

4. length of the literature item should be not less than 6 pages - shorter papers cannot convey enough information especially with respect to quality discussion and are irrelevant as concerns potential contribution to this research project (please refer to pilot validation of criteria in subsection 5.4.

First-Tier exclusion criteria are applied uniformly to extracted text corpus. Study can pass these set of criteria only if all criteria are fulfilled.

After initial quality screening and primary texts identifications, they are assessed to determine final text corpus. For this purpose, *Second-Tier inclusion criteria* are developed. These criteria are designed to differentiate relevant publications with the purpose to establish evidence on different data mining and data analytics methodologies and frameworks across different domains. As recommended by [4] these criteria are motivated by research scope (boundaries of research project), research objectives and questions.

Table 1 provide a summary of *Second-Tier criteria*.

Table 1. Second-Tier relevance criteria

Criteria Type and Number	Criteria Definition	Criteria Justification
Relevance 1	Is the study about data mining or data analytics approach and is within designated list of domains?	Exclude studies conducted outside the designated domain list. Exclude studies not directly describing and/or discussing data mining and data analytics
Relevance 2	Is the study introducing/describing data mining or data analytics methodology/framework or modifying existing approaches?	Exclude texts considering only specific, granular data mining and data analytics techniques, methods or traditional statistical methods. Exclude publications focusing on specific, granular data mining and data analytics process/sub-process aspects. Exclude texts where description and discussion of data mining methodologies or frameworks is manifestly missing

Quality Assessment As guided by [10], the next set of criteria (*Third-tier*) supports the author in differentiating studies into specific mutually exclusive categories with the defined spectrum/range. At the lowest end of spectrum are placed texts where methodologies and frameworks presentation and discussion is fragmented and up to the other end of spectrum where such presentation is executed in full. This is achieved by designing quality scoring metrics presented in Tabel 2 below.

Table 2. Scoring Metrics

Score	Criteria Definition
3	Data mining and analytics methodology or framework is presented in full. All steps described and explained, tests performed, results compared and evaluated. There is clear proposal on usage, application, deployment of solution in organization' s business process(es) and IT/IS system, and/or prototype or full solution implementation is discussed. Success factors described and presented
2	Data mining and analytics methodology or framework is presented, some process steps are missing, but they do not impact the holistic view and understanding of the performed work. Data mining process is clearly presented and described, tests performed, results compared and evaluated. There is proposal on usage, application, deployment of solution in organization' s business process(es) and IT/IS system(s)
1	Data mining and analytics methodology or framework is not presented in full, some key phases and process steps are missing. Publication focuses on one or some aspects (eg. method, technique)
0	Data mining and analytics methodology or framework not presented as holistic approach, but on fragmented basis, study limited to some aspects (e.g. method/technique discussion, etc.)

Screening procedures As mentioned above, *First-Tier quality* screening is executed by applying uniform set of criteria in text corpus extraction (language criteria, duplicates elimination, etc.) and in the process of working with the final publications corpus. As the study has passed these controls, it is assessed by Second-Tier criteria. Each criteria is judged from top to bottom. In principle, Relevancy criteria 1 and 2 are evaluated based on Abstract and Conclusion of the study. However, there are potential cases exempt from principles. In case of doubts or uncertainties on the content when assessing only Abstract and Conclusion, we are guided by inclusiveness on a safe side proceeding with evaluation based on overall publication text. By the same token, if clear decision cannot be reached, publication is passed to next evaluation level up until all criteria set is assessed. In case doubts persist even after full text analysis, publication is included in the final text corpus subject to analysis and synthesis stages (evaluation by *Third-tier Scoring metrics*). As detailed in the next section 5 Validation, criteria and screening procedures have been extensively piloted and confirmed.

5 Validation

Since protocol is critical component of the SLR, and given existing practice and recommendations (eg.[5]), two-fold validation procedure is designed and conducted. The first part involves intensive execution of pilot runs for data extraction, relevancy and screening process while the second is the formal protocol review by project supervisors. Here, the key aspects of the validation procedures and respective decisions and adjustments in the Systematic Literature Review method are summarized.

5.1 Search strings

Initially, we have tackled selection of the key words for the search strings and then ancillary elements to append to them. Key word "data mining" is determined by the research topic, however, we have to ascertain if the coverage and context of extracted text corpus is wide and balanced enough (external validity). As complementary key word, we opted to pilot and test "data analytics" term too[7]. The key motivation is as follows:

1. to be consistent with the observed research practices:
   – similar two-terms scope is defined extensively in the survey literature - in particular, we have noted the recurring pattern that surveys, addressing data mining consider both "data mining" and "data analytics" concepts (eg. [16]).
   – further, such two-term scope holds both for general domain-agnostic studies as well as particular domain-oriented literature (eg. [17]).
2. to meet the goals of the research - we have consulted number of practitioners' surveys (eg.[18]) and many of them reported the usage of various terms as well as degree of ambiguity with respect to usage of "data mining" and "data analytics" terms by practitioners. As the purpose of our SLR is to provide account on "state of practice" (including reference to pre-selected types of "grey" literature), inclusion of both terms will be beneficial.

By piloting "data analytics" and "data mining" terms on indexed database (Scopus) and non-indexed database (Google Scholar), we have confirmed that over the last decade: (1) "data analytics" term has started to be used much more extensively in the titles of publications and as key word (persistent pattern emerges starting from 2013), (2) on trial sample of publications we have confirmed extensive usage of both terms. Therefore, we have determined two key words "data mining" and "data analytics" to be used in search strings as final choice.

Initial choice of ancillary elements for search strings pilot has been guided by topic of the paper, we opted to include all standard terms used in the literature - "methodology", "method", "framework". The choice has been further refined as described below.

As mentioned in Section 4, initial search string combinations for 'data mining' keyword were ("data mining") AND ("methodology"), ("data mining") AND ("framework"), ("data mining") AND ("method"). Identical combinations were tested for 'data analytics' keyword too. However, piloting one of the searches on one of database (Scopus) revealed too wide scope with the more than 60 thousands publications retrieved just for one combination only. Therefore, the search string was adjusted to:

[7] The distinction between "data mining" and "data analytics" for example is very well-defined in [16]: "...by the data analytics, we mean the whole KDD process, while by the data analysis, we mean the part of data analytics that is aimed at finding the hidden information in the data, such as data mining".

– more narrow definition with strict combination, i.e. "data mining methodology", "data mining framework" and exclusion of "method" based on section 4.1 Scope decisions.
– search was restricted only to Title, Abstract, Keywords (TITLE-ABS-KEY) combination compared to initial search of All ITEMS.

In order to avoid omission bias and to confirm correctness of such decisions, sample of the first 40 most relevant hits for All ITEMS search was compared to Title+Abstract+Keyword search. It was confirmed that significant number of publications were retrieved identically for both searches. Moreover, the major difference with broader All ITEMS search originated from Proceedings compilations where the 'data mining' keyword was used for typically one article from the whole proceedings set; it usually focused on discussion of specific data mining case in the concrete scientific domain. Such information inclusion into scope would significantly increase retrieval and processing efforts, but would not contribute to providing more accurate or definitive answers to research questions.

5.2 Domains inclusion and exclusion criteria

As noted and similarly to [5], piloting has revealed that search engines retrieve literature available for all major scientific domains and naturally some part of scientific texts is published outside author's area of expertise (eg. medicine). Even though such studies could be retrieved, it would be impossible for authors to analyze and interpret correctly literature published outside the possessed area of expertise. The adjustments towards search strategy were undertaken by keeping areas closely associated with Information Systems, Software Engineering research while excluding areas not closely associated with these areas and also containing the least number of publications. Thus, for Scopus database the final set of inclusive domains was limited to 9:

1. Computer Science (no. of texts 597)
2. Engineering (291)
3. Mathematics (161)
4. Business, Management and Accounting (65)
5. Decision Science (64)
6. Economics, Econometrics and Finance (13)
7. Multidisciplinary (2)
8. Undefined (2)

Excluded domains covered 11.5% or 106 out of 925 publications and primarily focused on specific case studies in fundamental sciences or medicine:

1. Medicine, Biochemistry, Genetics and Molecular Biology
2. Environmental Science
3. Earth and Planetary Science, Physics and Astronomy
4. Energy, Material Science
5. Agricultural and Biological Science

6. Chemistry and Chemical Engineering
7. Pharmacology, Toxicology and Pharmaceuticals
8. Arts and Humanities
9. Neuroscience
10. Immunology and Microbiology
11. Health Professions and Nursing.

To confirm validity of such approach, two sets of experiments were conducted. Firstly, sample of 10 articles from Medicine domain publications was retrieved and analyzed. It was confirmed that set of publications is very specific, focuses on experimental research and its results interpretation, while applications and discussions on data mining methodologies are marginally manifested if at all. As additional factor, the excluded domain demonstrated rather limited number of publications with reference to data mining and data analytics compared to Computer Science and Engineering. This is not surprising given the fact that formal data mining methodologies originated from KDD (Knowledge Discovery in Databases) field [7]. Data mining methodologies have moved interdisciplinary with adoption in Medical, Chemistry, Physics and other research fields. However, the largest number of scientific publications on the topic have been naturally accumulated in the research area where data mining methodologies were originated and belong to. Secondly, the pilot search was executed removing restrictions with respect to search only on title, abstract and keywords (TITLE-ABS-KEY) and searching all items (ALL ITEMS) instead (please refer for detailed description subsection 5.1 above). The given search without domain restrictions yielded 3 902 results and with domain restrictions 3 479 excluding 423 items (10.8%) which is similar to domain adjustments described above.

5.3 Comparability of queries and their results across databases

As noted in [5], databases search engines are organized differently. Therefore, it was important to execute three types of checks and introduce respective adjustments if needed.

Firstly, the final search string combination constructed and piloted for Scopus database should produce similar results in case of Web of Science database, i.e. retrieval should be based on similar principles and logic. The pilot was run and first 20 items sample from both databases was compared. Part of publications retrieved were identical and even ranked on very similar relevancy level, overall samples matched with respect to research scope and content. Therefore, it was confirmed that constructed queries were comparable and applicable for Scopus and Web of Science domain without specific adjustments.

Secondly, Scopus search was constructed limiting to Title, Abstract and Keyword search. Web of Science has different taxonomy of potential search items. The correct and fully corresponding match was found to be Topic search which by default includes Title, Abstract and Keywords.

The third comparability should have been ensured with respect to research domains included and excluded from scope. Not surprisingly, Web of Science

search engine has more granular taxonomy of research areas than Scopus. The corresponding match for both taxonomies was performed, in case of doubts the cautious approach was applied on a safe side of inclusiveness.

5.4 Minimum length inclusion criteria

The protocol limited length of the literature item to not less than 6 pages. To prevent omission bias, sample of 10 articles with less than 6 pages retrieved from Scopus database as well as Google Scholar were checked. It was confirmed that set criteria is not restrictive with respect to valuable information, analyzed studies were on average 3-4 pages, have been either primarily "grey" literature texts or originating from publication sources which reliability and traceability (eg. if the source is peer-reviewed) has been difficult to identify. In terms of content, the short studies either presented high-level overviews and compilations with very limited or no discussions or alternatively specific case study with the focus on results reporting and interpretations. Therefore, minimum length criteria was confirmed.

6 Conclusion

This protocol has presented planning and validation procedures for Systematic Literature Review. The latter will be conducted within PhD research project on data mining and data analytics methodologies and frameworks in financial services domain. Importantly, the plan is based on best practices and recommendations primarily adapted towards and currently used in Information Systems and Software Engineering research.

The presented outcome of the executed planning phase are motivated and documented SLR objectives, process and detailed method including scope, search strategy and screening principles. Authors also developed two-tier quality and relevancy criteria decision tree to execute publications screening as well as Scoring metrics to evaluate quality of publications. Based on the best practices and recommendations, protocol was also intensively piloted ensuring validation and thorough exploration of all key elements of SLR prior to starting its execution. Authors believe that proposed process design and executed validation procedures minimize potential biases and ensure adequate transparency, traceability and replicability of the research process and its outcomes.

Declarations

Conflicts of interest: There is no conflict of interest for the author

Contributorship and review: The protocol was written by V. Plotnikova (PhD student undertaking and executing it as part of broader PhD project at Institute of Computer Science at University of Tartu). Validations procedures have been performed by Veronika Plotnikova too. Protocol was iteratively reviewed, discussed and approved by supervisors of PhD project Prof. Marlon Dumas and Fredrik P. Milani.

References

1. Nasdaq Globe Newsire, Nashua, N., H., Dresner Advisory Services Publishes 2017 Big Data Analytics Market Study, available at https://globenewswire.com/news-release/2017/12/20/1267022/0/en/Dresner-Advisory-Services-Publishes-2017-Big-Data-Analytics-Market-Study.html, last accessed 2019/10/26
2. Forbes, Louis Columbus, 53% Of Companies Are Adopting Big Data Analytics, Dec 24 2017, available at https://www.forbes.com/sites/louiscolumbus/2017/12/24/53-of-companies-are-adopting-big-data-analytics/4cf12a2139a1, last accessed 2019/10/26
3. Digital Journal, James Walker, available at http://www.digitaljournal.com/tech-and-science/technology/big-data-strategies-disappoint-with-85-percent-failure-rate/article/508325, last accessed 2019/10/26
4. Kitchenham, A. Barbara, Budgen., D., Brereton., P.: Evidence-based software engineering and systematic reviews. Vol. 4. CRC press, (2015)
5. Brereton. P., Kitchenham, A. Barbara, Budgen., D.,Turner, M., Khalil, M.: Lessons from applying the systematic literature review process within the software engineering domain. Journal of Systems and Software **80**, 571–583 (2007)
6. Vanwersch, R.J.B., Shahzad., K., Grefen, W.P.J., Pintelon, L.M., Mendling, J., van Merode, G.G., Reijers, H.A: Methodological support for business process redesign in health care: a literature review protocol. International Journal of Care Pathways **15**, 119–126 (2011)
7. Fayyad, U., Piatetsky-Shapiro, G., Smyth, P.: The KDD process for extracting useful knowledge from volumes of data. Communications of the ACM **39** (11), 27–34 (1996)
8. Hevner, R.A., March, T. Salvatore, Park, J., Ram, S.: Design Science in Information System Research. MIS Quarterly **28** (1), 75–105 (March 2004)
9. Kofod-Peters, A.: How to do a Structured Literature Review in Computer Science. Technical Report, published https://www.researchgate.net/publication/265158913 (October 2014)
10. Kitchenham, B., Charters. S: Guidelines for performing systematic literature reviews in software engineering. Technical Report, EBSE Technical Report EBSE-2007-01, (2007)
11. Schöpfel Joachim: Towards a Prague Definition of Grey Literature. Twelfth International Conference on Grey Literature: Transparency in Grey Literature. Grey Tech Approaches to High Tech Issues.Prague, 6-7 December 2010, Czech Republic, 11–26, (Dec 2010)
12. Vahid Garousi, Michael Felderer, Mika V. Mäntylä: The need for multivocal literature reviews in software engineering: complementing systematic literature reviews with grey literature. EASE 2016: 26:1-26:6
13. Vahid Garousi, Michael Felderer, Mika V. Mäntylä: Guidelines for including grey literature and conducting multivocal literature reviews in software engineering. Information and Software Technology (106), 101–121, (2019)
14. Neto, G. T. G., Santos, W. B., Endo, P. T., and Fagundes, A. R.: Multivocal literature reviews in software engineering: Preliminary findings from a tertiary study. In 2019 ACM/IEEE International Symposium on Empirical Software Engineering and Measurement (ESEM), 1–6, IEEE, (Sep 2019).
15. Yasin, A. and Hasnain, M.J.: On the quality of grey literature and its use in information synthesis during systematic literature reviews, Blekinge Institute of Technology, School of Computing, Master Thesis no. MSE-2012:97, Software Engineering (Sep 2012)

16. Tsai, C.W., Lai C.F., Chao H.C., Vasilakos A.V., Big data analytics: a survey. Journal of Big data. 2(1):21, (Dec 2015)
17. Archenaa, J., Mary Anita, E.A.: A survey of big data analytics in healthcare and government. Procedia Computer Science 50, 408–413, (2015)
18. Russom P.: Big Data Analytics. TDWI best practices report. TDWI Research, fourth quarter, (2011)
